# Towards implementing artificial intelligence post-processing in weather and climate: proposed actions from the Oxford 2019 workshop

**DOI:** 10.1098/rsta.2020.0091

**Published:** 2021-02-15

**Authors:** Sue Ellen Haupt, William Chapman, Samantha V. Adams, Charlie Kirkwood, J. Scott Hosking, Niall H. Robinson, Sebastian Lerch, Aneesh C. Subramanian

**Affiliations:** 1 Research Applications Laboratory, National Center for Atmospheric Research, Boulder, CO, USA; 2 Scripps Institute of Oceanography, La Jolla, CA, USA; 3 Met Office Informatics Lab, Exeter, UK; 4 Statistical Science, University of Exeter, Exeter EX4 4QE, UK; 5 British Antarctic Survey, The Alan Turing Institute, London, UK; 6 Met Office Informatics Lab, University of Exeter, UK; 7 Karlsruhe Institute of Technology, Karlsruhe, Germany; 8 Atmospheric and Oceanic Sciences, University of Colorado Boulder, CO, USA

**Keywords:** artificial intelligence, machine learning, weather, climate, post-processing

## Abstract

The most mature aspect of applying artificial intelligence (AI)/machine learning (ML) to problems in the atmospheric sciences is likely post-processing of model output. This article provides some history and current state of the science of post-processing with AI for weather and climate models. Deriving from the discussion at the 2019 Oxford workshop on Machine Learning for Weather and Climate, this paper also presents thoughts on medium-term goals to advance such use of AI, which include assuring that algorithms are trustworthy and interpretable, adherence to FAIR data practices to promote usability, and development of techniques that leverage our physical knowledge of the atmosphere. The coauthors propose several actionable items and have initiated one of those: a repository for datasets from various real weather and climate problems that can be addressed using AI. Five such datasets are presented and permanently archived, together with Jupyter notebooks to process them and assess the results in comparison with a baseline technique. The coauthors invite the readers to test their own algorithms in comparison with the baseline and to archive their results.

This article is part of the theme issue ‘Machine learning for weather and climate modelling’.

## 1. Background

Artificial intelligence (AI) and machine learning (ML) show promise for improving modelling and forecasting for a host of problems. Environmental science is one of many applications of this useful technology [1]. Although weather and climate have been traditionally modelled using dynamical and physical models built from first principles, more empirical methods have also proven useful; thus, it is natural that AI/ML would find applications in this field. Hereafter, we will use AI to encompass ML in our terminology.

A workshop on Machine Learning for Weather and Climate was convened at Oxford, UK, in September 2019 to assess the state of the science, evaluate progress, and propose next steps along the pathway to realize the potential of AI in the atmospheric sciences. Some of the first lectures segmented the research broadly into three primary groups: post-processing, emulating processes and using ML to build full models [2,3].

The workshop provided time and space to discuss each of these topics more broadly. Most of these coauthors became part of the working group assessing opportunities for AI to improve the output of environmental science models, known as post-processing, while the rest contributed to the on-going conversation and effort to archive datasets to help advance the science. This group not only assessed the successes of the environmental science community in leveraging AI for post-processing to date but also discussed the importance of disclosing failures as a measure to help advance the science more rapidly. The authors believe that a vigorous effort should be made to explore and validate modern AI methods. We see a host of opportunities to further improve numerical weather prediction (NWP) forecasts and climate projections at a minimal cost when compared with other model development efforts. We suggest what is needed to move forward, discuss what will constitute success and make some concrete recommendations for the next steps, including beginning an archive of example problems that can be used to test emerging methods.

Model post-processing corrects systematic errors in model output by comparing hindcasts to observations. This is becoming increasingly important, but also challenging, as NWP model resolution has increased to the point that it attempts to resolve hyper-local effects and structures with a stochastic nature. Similarly, in climate projections, there is a drive towards more localized information, which is inherently uncertain. For context, NWP forecast systems, through model improvements and assimilation of additional observational data, have historically achieved a root-mean-square error (RMSE) skill improvement of approximately one day every 10 years [4]. However, this skill has arguably been attributable to increases in supercomputing power that has enabled higher model resolution and more comprehensive data assimilation [5]. Unfortunately, this progress is unlikely to continue under the death of Moore's Law (https://www.nature.com/news/the-chips-are-down-for-moore-s-law-1.19338). In this context, model post-processing becomes yet more important to help drive skill improvements at uncertain length-scales and with ever more limited compute resources. AI is a prime candidate for developing more powerful post-processing approaches that can represent cheap transfer functions in fractions of the development time of traditional approaches (e.g. [6]).

AI also brings the ability to optimize output for specific tasks by choosing appropriate loss functions. The same set of NWP forecasts may be post-processed in different ways according to the needs of particular end-users and the decisions they have to make. Built on statistical foundations, AI post-processing systems not only have the capability to correct biases and phase shifts in numerical forecasts, but also have the potential to quantify forecast uncertainty—both epistemic (due to lack of knowledge) and aleatoric (natural randomness of a process)—more comprehensively than NWP approaches, and in doing so provide better information for decision support. In this sense, AI post-processing can act as a bridge between the physical representation of the atmosphere provided by NWP and the decision-making requirements of end-users. One must also recognize the observation error in the ‘truth’ data to which the AI is trained. If that error is systematic, AI will often discover and correct it. Even if that error is aleatoric, AI can learn a correction on average to minimize the error.

This manuscript reports on the state of the science of AI post-processing for weather and climate and provides a foundation for further progress through recommending a repository of methods and data that can enable the community to move forward. Section 2 provides a brief history of the development and use of AI for post-processing weather and climate model output without attempting to be comprehensive. The working group considered the current challenges and how the community might most effectively address them, including setting some medium-term goals as discussed in §3. Section 4 considers what successful application of AI post-processing in weather and climate will look like. The workshop attendees decided to make a distinct impact through concrete deliverables as laid out in §5. In particular, we describe the need for a repository to provide common assessment tools and datasets for ML scientists to test methods and set the stage for intercomparison. The repository and initial datasets are described. Section 6 summarizes and provides some concluding thoughts.

## 2. Emergence of AI post-processing—A brief literature review

Although the dynamic models of weather and climate have formed the basis for prediction, the community has long recognized the value of post-processing the forecasts to improve accuracy and to quantify uncertainty.

Global Climate Models (GCMs) and NWP models provide the atmospheric variables necessary to determine predicted atmospheric states based on numerical integration of a discretized version of the Navier–Stokes equations [7]. However, due to uncertainty in initial conditions and numerical approximation as well as the non-linearity of the system, the chaotic error tends to swamp skill from initial information [8]. In addition, model deficiencies add systematic error and insufficient observations put a limit on the resolution of initial conditions. For as long as NWP forecasts have been officially issued there have been attempts to statistically correct these methods, given observational data (e.g. [9]). This can be viewed directly as a supervised machine learning task. Current weather forecasting centres, including the UK Met Office, US National Center for Environmental Prediction (NCEP), European Center for Medium-Range Forecasting (ECMWF), and many others rely on statistical methods that have been proven successful. The initial methods employed multilinear regressions and became known as Model Output Statistics (MOS [9]). These systems expanded to treat ensembles and became Ensemble Model Output Statistics (EMOS) [10,11]. These statistical learning methods have been used in practice since 1968 by the U.S. National Weather Service to improve systematic model error (e.g. [12,13]). These methods are continually refined with new observational data and show major skill improvement in correcting forecasts from 0 to 10 days [14]. MOS and EMOS, however, are inherently linear techniques that are notoriously rigid and require significant tuning (e.g. specification of predictive distribution and estimation of the parameters, e.g. the mean and the standard deviation in the case of a Gaussian distribution). AI methods, which typically allow for the resolution of complex nonlinear processes, open up opportunities for more effective corrections.

### (a) Forecast improvements with AI

**Figure 1.**
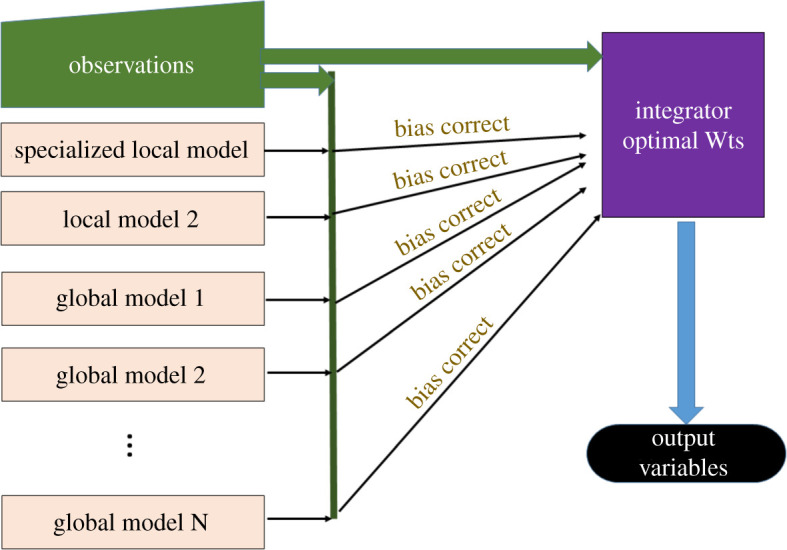
DICast post-processing progresses in a two-step process using historical forecasts: 1) bias-correcting each model's input using any of a number of MOS-like methods and 2) determining optimal weighting for each model for each forecast time and each lead time [15]. (Online version in colour.)

Despite the success of statistical corrections, weather forecasting has a tradition of human forecasters weighing the relative merits of the various models according to the situation. AI came into play as the private sector began to forecast beyond a single country, and it became obvious that human forecasters could no longer do corrections from experience for the entire globe. The Weather Company realized this in the late 1990s and collaborated with the National Center for Atmospheric Research (NCAR) to develop the Dynamic Integrated foreCast system (DICast®) that learns the appropriate weights for input models given paired historical forecasts and observations [15]. Figure 1 demonstrates the DICast post-processing methodology, which is representative of the many other systems currently being used. DICast has evolved over time to include additional machine-learning methods and has been shown to dramatically improve forecasts across multiple weather-dependent applications including road conditions [16], precision agriculture, wind and solar energy [17–20], among others. Now, many commercial weather companies and national centres employ AI-based post-processing methods [21].

To deal with the aforementioned inherent uncertainty in NWP forecasts, the national centres now run ensembles of model forecasts with perturbations to initial conditions or other methods of initiating perturbations in the simulations [22–25]. As with deterministic forecasts, these ensemble forecasts may have biases in their mean and the spread may not be calibrated against the actual uncertainty. Thus, methods to ameliorate these problems have been devised to bias correct the mean deterministic value as well as to calibrate the spread. The first techniques developed were statistical methods such as the EMOS described earlier [10,11], quantile regression [26,27], Bayesian model averaging [28], linear variance calibration [29,30], among others. This challenge also can be met through the application of AI methods. Some of these methods involve identifying regimes using some clustering or another method to identify similar past forecasts. Hamill and Whittaker [31] and Hamill *et al*. [32] describe an analogue approach to calibrating ensembles of precipitation forecasts. Greybush *et al*. [33] describe a multi-step approach that first splits the forecasts into regimes using principal component analysis then applies AI-based methods to distinguish among weather regimes to produce weighted consensus forecasts of surface temperature. McCandless *et al*. [34] tested multiple AI methods for improving ensemble member weighting for predicting snowfall accumulation.

Several methods have been used to more directly provide probabilistic information with AI approaches. Krasnopolsky [35] reviews the use of Artificial Neural Networks (ANNs) to form ensembles for various applications. He compares nonlinear ANN approaches to linear ones and demonstrates marked improvements using the nonlinear approaches for several variables. Evolutionary programming (EP) has also proven useful for generating AI ensembles. EP methods have been used to evolve ensembles, demonstrating that smaller temperature RMSEs and higher Brier Skill Scores could be generated than with a 21-member operational ensemble [36]. This method is also useful for minimum temperature forecasts, then demonstrated further improvements for adaptive methods [37,38]. The analog ensemble (AnEn) method has arisen as a machine-learning technique to directly predict both deterministic forecast values and to quantify uncertainty directly from a single high-quality NWP run of sufficient length [39]. The AnEn uses a time series of historical forecast variables and their corresponding verifying observations. For each current forecast being made, the AnEn looks back in the historical record to find the *n* most similar forecasts. The verifying observations then become an *n-*member ensemble that is used to estimate the uncertainty of the forecast. The mean of that ensemble becomes the improved forecast value. The AnEn has been shown to improve upon raw ensemble output as well as upon some common statistical methods [39].

### (b) Applications driving post-processing

AI methods have been highly used in applications that derive from NWP forecasts. For instance prediction of severe weather has seen a plethora of AI methods applied to improve predication [40]. Random forests [41], for instance, can model nonlinear relationships including arbitrary predictors while being robust to overfitting. In weather post-processing, quantile regression forest models have been proposed by Taillardat *et al*. [42] and extended to include combinations with parametric approaches [43]. The prediction of mesoscale convective areas has been shown to be successful with decisions trees by Gagne *et al*. [44] and Ahijevych *et al*. [45]. Gradient boosted regression trees proved the most accurate method for predicting storm duration and forecasting severe wind [46]. Gagne *et al*. [47,48] have applied machine learning methods including random forests and gradient boosted regression to predict the probability of severe hail.

Where applications have financial implications may be where AI has been applied most frequently to improve forecasts. For instance, as more renewable energy is being deployed, it becomes increasingly important to accurately predict the daily variations in wind speed and solar irradiance directly at the plants using local observations. AI combined with NWP models has proven to be a best practice to estimate the timing of changes [17,19,49]. Methods such as autoregressive models, Artificial Neural Networks, Support Vector Machines, and blended methods have shown success at providing nonlinear corrections to models [15,50,51]. Such techniques can improve upon a forecast by 10–15% over the best model forecast [15,19,49]. Probabilistic forecasts of these variables are also important to industry [52–54]. The AnEn described above has proven useful for predicting both wind [55] and solar power [56].

### (c) Longer time scales

Beyond the NWP forecasting timescales of 10–14 days, forecast centres are increasingly providing subseasonal and seasonal-scale forecasts. At seasonal to decadal timescales, initialized coupled climate models are used to skilfully forecast shifts in regional climates [57]. However, this skill relies on extensive post-processing to correct for regression from initialized climatology to model resting climatology known as ‘bias-correction’ [58].

A growing area of research is to use known knowledge of physics in terms of physical laws and conservation properties in machine learning algorithms to constrain the training and improve the algorithms further. For example, a generative adversarial network (GAN)-based model for simulating turbulent flows can be further improved by incorporating physical constraints, e.g. energy spectra [59], in the loss function. Convolutional neural network (CNN)-based models for parameterizing subgrid-scale physics can be further improved to represent the mean climate by constraining global conservation properties e.g. conservation of momentum [60].

Climate model simulations routinely have spatial and temporal (e.g. seasonal) biases with respect to observations, some of which are systematic across climate models (e.g. Southern Ocean warm bias as described in [61,62]). When constructing future climate predictions using available simulations run under set future emission scenarios, it is important that any model biases are corrected before making impact assessments (e.g. crop yield projections which may be a function of the number of days above/below a given threshold within the growing season). These bias corrections are often made by calculating the differences (deltas) in probability distribution functions (PDFs) between the historical climate model and observations and then applying these deltas to the future climate simulation. This assumes that the biases are not time-varying. Similarly, another post-processing method known as change factor calculates the PDF deltas between historical and future climate runs within the same climate model and then applies these deltas to the observed distribution. Depending on the shape of the climate variable's distribution (Gaussian versus skewed), the PDF deltas may be calculated by using the PDF means, means and variance, or quantile mapping [63,64]. There is great potential here to use AI to perform nonlinear multivariate and spatial-temporal bias correction on climate model output.

A similar process was applied to assess the changes in the wind and solar resources over the United States in a projected climate change scenario for a period spanning 2040–2069 based on GCM simulations [65]. In that work, self-organizing maps (SOMs) were used to distinguish patterns representative of climate regimes, then to simulate a proxy future climate through Monte Carlo simulation of the correct pattern for a given month in the future, utilizing a computed bias correction (similar to the change factors) for future temperature changes.

### (d) Deep learning for forecast improvement

More recently, deep learning (DL) techniques have been revolutionizing how spatial data can be analysed and better predicted. DL neural networks and their subclasses (convolution, long-short term memory, etc.) are known to be able to approximate nonlinear functions [66] with the developed transfer functions learned from the data alone. Gagne *et al*. [48] have applied convolutional neural networks (CNN) to NWP data to identify storms most likely to develop severe hail, then to identify features of those storms that make them hail producing. Lagerquist *et al*. [67] used CNN to predict the movement of weather fronts, and Chapman *et al*. [68] showed these methods to be superior for predicting integrated water vapour, an indicator of atmospheric rivers. McGovern *et al*. [69] demonstrated how to advance beyond just blindly applying these methods to better understand physics. These methods have proven successful for deterministic forecast improvement that encodes spatial information while also being able to provide tuned probabilistic estimates of uncertainty (e.g. [6,70]). Gronquist *et al*. [71] demonstrate applying DL methods to substantially improve uncertainty quantification skills for global weather forecasts, including for extreme weather events.

### (e) Beyond post-processing and toward decision-making

In addition to the use of AI for post-processing individual weather models, there is an opportunity, and perhaps a need, to use AI as an ‘algorithmic interface’ to weather model output. As ever more weather models come online, each with increasingly high resolution and with more numerous ensemble members, meteorologists are increasingly stretched to reliably and accurately summarize the available information into meaningful forecasts for end-users. While this is less of an issue in day-to-day forecasting (where, for example, reporting a simple ensemble mean—human out of the loop—may be sufficient) it becomes much more significant in the context of hazard warnings, where probabilistic forecasts need to be well-calibrated in order to be effective.

In fact, there exists a gap between the information output by NWP models (a set of predictions of weather outcomes, each likely to be carrying biases) and the information required to make optimal decisions (which, according to decision theory, would be a well-calibrated probability distribution over weather outcomes). While the outputs of traditional NWP modelling could be viewed as a sparse approximation of the desired probability distribution over outcomes, the use of AI to debias and ‘infill’ this probability distribution based on all available information seems an important area for AI post-processing. The development of such systems, which can optimally extract and present information from the range of models they oversee, has the potential to not only improve on probabilistic forecasting when it matters most but also facilitate individual model development by providing overarching consistency in output, so that drastically changing an individual model will not break the system (the overall output will be carried by the other unchanged models until the performance of the updated model is sufficiently well learned to be given influence). This behaviour could be achieved through dynamically weighing the influence of the separate forecasting models, according to a model stacking procedure (e.g. [15]). This ‘AI overseer’ approach also opens the door to use more experimental forecasting model designs in operational settings, for example, purely statistical forecasts could be run alongside numerical ones, with their optimal weightings in the final output learned dynamically on-the-fly.

### (f) Cost value of AI post-processing

As with any method, there is a cost to modelling that involves obtaining sufficient amounts of data, computational time and researcher time. These costs vary widely depending on the task to be performed, the data requirements, the method employed, and the accuracy desired. Although there are rules of thumb for data requirements for some methods, there are many exceptions to those rules. For instance, for a simple temperature forecasting post-processing method with an ANN, one typically desires at least a year's worth of data to capture diurnal and seasonal cycles in the data (one may wish to include day-of-year and hour-of-day variables). With multiple years of data, accuracy may improve. These data requirements are not dissimilar to those of statistical methods such as MOS. For dynamic methods such as DICast that are retrained frequently, less data may be required to produce optimal results—DICast can be optimized with 90 days of data or less [15,20]. The computational time for these methods is trivial in comparison with the time to accomplish the NWP simulations. Standard applications have become rather inconsequential in terms of required person time to train, test and apply the methods. Research into how to design optimal methods, such as any research problem, can consume as much personal time as the researcher has interest. In contrast, deep learning problems with many inner nodes require substantially more data and computational time to train the DL model.

One of the few examples of a cost/benefit analysis of an AI application was accomplished by Delle Monache *et al*. [39] who trained an AnEn on a single high-quality NWP simulation and compared it to running a coarser resolution 21-member ensemble with EMOS post-processing. They found that the AnEn performed better in terms of both deterministic and probabilistic forecasts at a substantially lower computational cost.

## 3. What is needed to move forward

We see an expeditious and successful post-processing AI and ML community being predicated on four features: trustworthiness, interpretability, usability and technique.

### (a) Trustworthiness

Since AI is now being used across many domains for decision-making that affects people's lives, there is growing realization by funding bodies that trustworthiness is a key factor in the continued uptake of such systems. For example, in the UK, UKRI has already established doctoral training centres for ‘Accountable, Responsible and Transparent AI’ and ‘Safe and Trusted Artificial Intelligence’ (as examples, ref UKRI website: https://www.ukri.org/research/themes-and-programmes/ukri-cdts-in-artificial-intelligence/, and in the US https://www.technologyreview.com/2020/01/07/130997/ai-regulatory-principles-us-white-house-american-ai-initiatve/). In scientific domains, robustness and reproducibility are important factors that influence trust. In the past, AI research has often ignored these factors, but the community is becoming more aware of the issues. For example, some recent studies have attempted to apply greater rigour to benchmarking and comparing similar techniques [72] and also proper evaluation of the claim that metric learning systems have been achieving ever-increasing accuracy [73]). These studies found various deficiencies such as the way algorithms were compared, poor training and hyperparameter tuning strategies and weaknesses in accuracy metrics. In particular, Musgrave *et al*. noted that the AI community lacked proper benchmarking strategies. Direct and interpretable method success and failure metrics are crucial for impactful and trustworthy post-processing methods. In practice, this means testing against classic techniques (MOS, EMOS, Bayesian model averaging, etc) to determine the level of effectiveness of the proposed methodology with rigorous confidence intervals (i.e. block bootstrapping) on data that the method and the practitioner have not previously used. This includes separating the training, testing, and validation data into temporal slices to ensure that no temporal correlation can cause artificially inflated skill between testing and training. Standard techniques exist in the weather community to evaluate both probabilistic (continuous ranked probability score, rank histograms, etc.) and deterministic (RMSE, bias, correlation, etc.) skills. However, the correct metrics must be chosen for the target variable. For example, RMSE can be largely ineffective for precipitation, a field dominated by null values and can lead to erroneous results, where thresholded relative operating characteristics might be much more appropriate. Rigorous and tedious testing will help to ensure each method's worth and elucidate the true value added by the post-processing.

### (b) Interpretability

Related to the previous section, trust in AI methods is also affected by a lack of interpretability due to the complex structure of typical AI architectures. AI is plagued by the so-called ‘black box’ syndrome, although this perception is often quoted without domain knowledge. In response, a scientific effort has emerged to demystify the inner workings of the AI methods and instill community-wide trust in their use [74–76]. More recently, the environmental sciences community has also taken up this challenge and is striving to develop trust and acceptance around AI interpretability and to demonstrate an understanding of the underlying physics at play [69,77]. This emphasis on explainable AI is beginning to resonate with the funding agencies, which is now accelerating research in this area. For instance, specific success has been seen in interpretable machine learning in the weather community, including Jacobian methods of saliency, backwards optimization and class activation [48,67], and input permutation for feature importance [6,41,78]. Another area revolves around novelty detection in conjunction with principal component analysis [79].

### (c) Data usability

A statistical post-processing task begins with data cleaning. This process is often unnecessarily tedious owing to the structure and unique ‘edge-cases’ inherent to output model data. Clear documentation and use cases in the output forecast file would expedite the cleaning process exponentially. This includes metadata and any processing (regridding, averaging, etc.) that has been performed on a given dataset, including missing values and the accurate date and time stamps. We recommend that modelling centres adopt the ‘FAIR principles’ ([80]; https://www.go-fair.org/fair-principles/), namely data must be 1) Findable, 2) Accessible, 3) Interoperable and 4) Reusable.

Machine and statistical learning require long and consistent datasets without shifting systematic distribution relationships between forecasted and observed conditions. Thus, new model development is detrimental to the post-processing techniques. Experiments have shown that two seasons of homogeneous data (approx. 300 forecasts) are required to elucidate stable statistical biases using traditional linear approaches [12], while bootstrap experiments with surface wind data indicated that more than 200 cases would be required to control overfitting of the development sample [81]. Linear methods (like MOS) could potentially benefit from this short of a training set, but deep learning methods require much more data to develop the conditional bias relationships that we hope to discriminate. We, therefore, urge that each new model development system creates and retains long historical reforecast data sets. These reforecasts should be planned as part of the iterative model improvement and release cycle. To that end, we call for a systematic study of reforecast length versus post-processing skill in order to more accurately capture the required length of reforecast data. The question then becomes: is it more valuable to develop better NWP or better post-processing? How should weather services balance their efforts and weigh the potential improvements from additional training data against potential improvements from NWP model improvements [82]? Additionally, we should consider developing and assessing modelling systems by the skill of the post-processed model output, rather than that of the model alone.

All supervised AI post-processing techniques require ground truth data to develop a linking function between the forecast and observations. The continued development of new long-running reanalysis products [83] provides many desired ground truth variables (i.e. temperature and precipitation). However, less common ground truth variables are often tedious to calculate due to massive data download requirements. The post-processing workflow could be expedited if modelling centres continued communication with end-users about desired labelled output variables. Efforts by modelling centres are already underway to integrate user feedback and produce desired variables (i.e. lightning, integrated vapour transport and Max CAPE/CAPES https://www.ecmwf.int/sites/default/files/elibrary/2018/18260-ecmwf-product-development.pdf), and the authors commend and encourage this collaboration.

In order to further develop successful techniques, weather benchmarking datasets need to be developed and curated for fast technique development. Standardized datasets that have been post-processed with classic methods should be made available to the community to quickly test the efficacy of new ideas and methods. Such datasets will enable rapid prototyping and architecture testing.

Lastly, research may demonstrate the enhanced skill of a new technique, but will that skill be successfully realized in an operational system? Thus, engineering a post-processing library is vital for proper technology transfer. Additionally, communicating early with the intended end-user to determine needs will expedite the entire process. Finally, the movement toward a culture of sharing code and model implementations could push science forward at a much faster rate.

### (d) Technique

The AI community constantly develops new modelling methods to capture as much predictable skill from a dataset as possible. The key for the weather community is to leverage domain knowledge to determine what in these new methods is appropriate and valuable for weather forecast post-processing. For example, Chapman *et al*. [68] leveraged convolutional neural networks, which develop spatial relationships acting on input image data, to capture large-scale weather features (rather than local forecast features alone) for predictive point measurement post-processing.

Due to the aforementioned sensitivity to initial conditions, uncertainty quantification has become a priority of forecasting centres. Thus, the major forecasting centres rely on ensemble systems in order to capture the uncertainty inherent in the natural variability of the weather system and model initialization. The rise of Bayesian ML methods (Gaussian processes, etc) and Bayesian neural networks, which produce distribution-to-distribution regression, can help quantify the uncertainty in a post-processed value rather than predict the mean state alone. Other methods are reviewed in §2. Perhaps, we could replace model ensembles with ML post-processing and substantially decrease the required computational resources by eliminating some ensembles. Lee *et al*. [84] showed that for forecasting 2-m temperature and 10-wind, with calibration the number of members of an NWP ensemble could be cut in half. Subsequent work indicated that the weighting of ensemble members varies by season, but that a 42-member physics ensemble could be represented with just 7–10 members [85]. Such an approach would allow computer power to be devoted to higher resolution NWP simulations in place of more ensemble members. This work requires further testing but offers an exciting avenue for probabilistic forecasting. Parallels can be drawn between this thinking and the popular AnEn methods, which produce well-calibrated and unbiased ensemble estimates from a single NWP simulation [39].

## 4. What will constitute success?

The working group considered major goals as metrics for success in the coming years. For the weather community, successful use of AI will be visible when major centres include AI post-processing as a step in how they make their forecasts. Several centres are moving in this direction. For instance, Météo France is currently implementing a random forest for post-processing ensemble forecasts [26], paving the way towards more full implementation.

For AI to be fully integrated, this would imply that when changes are made to the systems, the centre would consider the post-processed result rather than the output of the NWP models alone. It would also involve making computational space and time for the AI method a priority. This would also imply trust in the methods, which will come with rigorous statistical validation. Such applications could be in terms of post-processing NWP output, ML downscaling, implementation as part of satellite products, enhancing prediction for high impact events and anomaly detection. It may involve conditional correction, such as identifying a regime and providing regime-dependent corrections. To accelerate such progress requires the ability to not only publish successful applications but also the failures. If failures are also routinely published, a vast amount of time could be saved by not having each research group try the same thing. In fact, a repository of failures could be quite valuable to the community.

In the climate arena, downscaling using AI could save vast amounts of computational power and time while maintaining the type of accuracy needed if research advances to the place where the methods are fully trusted. AI can assist with intelligently weighing the models in CMIP runs to produce a ‘best estimate’ rather than a simple mean or median. Using feature detection as a new product of the output could aid in better understanding changes in patterns and the potential emergence of new patterns.

As discussed in the prior section, community trust in the output of AI-post-processed model runs could lead to faster discovery and deeper understanding of weather and climate simulations. This acceptance hinges entirely on the development of interpretability methods and statistically rigorous proof of model improvement.

## 5. Actionable items

To achieve the vision articulated above, some specific actions could form a roadmap to catalyse the application of AI post-processing towards achieving the vision articulated above. Specifically, we call for 1) development of a data repository for fast development of post-processing techniques, 2) data standardization methods (FAIR), 3) calls for studies on interpretability methods, 4) metadata and model documentation for labelled training data and 5) a database of recorded AI failures to limit duplication of effort across the research community.

As a result of these deliberations, we wish to contribute to the actions that we propose. In particular, we propose an open-access experimental testbed database on which traditional methods have been implemented in order to set benchmarking points for the rapid development of new machine learning methods [86]. We have chosen these datasets to represent various temporal and spatial scales and problems that are of current interest to atmospheric scientists. To initiate this repository, we provide five separate and clean weather and climate forecast fields (detailed below) from over eight modelling agencies, along with the verifying forecast values. The datasets include both ensemble and deterministic forecasts and offer a plethora of avenues for post-processing research. The data are permanently archived at the University of California San Diego Libraries (https://doi.org/10.6075/J08S4NDM), and we provide tested Python code to aid in rapid analysis and evaluation of results (https://github.com/NCAR/PostProcessForecasts). Each dataset includes truth data, model data, and an example application. The problems are summarized in table 1.

**Table 1.** Summary of five datasets archived in repository.

Data Set	Madden-Julian Oscillation Forecast	Pacific North American Forecast	Integrated Vapour Transport (IVT) Forecast	Germany T2 m Forecast	UK Surface Road Conditions Forecast
Modelling Center	CMA, CMC, CPTECT, ECMWF, JMA, KMA, NCEP, UKMO	CMA, CMC, CPTECT, ECMWF, JMA, KMA, NCEP, UKMO	NCEP–GFS	ECMWF	UKMO–MORST
Forecast type	Ensemble	Ensemble	Deterministic	Ensemble	Ensemble
Forecast lead time	0–15 days (daily)	0–15 days (daily)	006 h, 048 h, 168 h	48 h	0–168 h (hourly)
Region of interest	Combined EOF 1 & 2 of 15° S–15° N average U200 & U850 as in (Wheeler and Hendon 2004 [87])	PNA Lat/Lon locations as in (Wallace and Gutlzer 1981 [88])	Gridded Lat[10° N, 60° N], Lon[180°,110° W] (0.5° × 0.625°)	537 German observation station locations	Four undisclosed locations
Time span	2006–2019	2006–2019	2006–2018	2007–2016	Dec 2018–Mar 2019
Ground truth	Forecast hour 0 RMM1 and RMM2 index analysis	Forecast hour 0 PNA index Analysis	Gridded MERRA2 reanalysis IVT (0.5° × 0.625°)	T2 m stations	Station surface temperature
Variable of Interest	RMM1 and RMM2 Index Forecast	PNA Index Analysis	Gridded GFS IVT forecast (0.5° × 0.625°)	T2 m Forecast	Road surface forecast

The Github repository provides a series of Jupyter Notebooks demonstrating how to load, interpret, prepare and split datasets, train simple benchmark post-processing algorithms and score the output with appropriate scoring metrics typical within the weather forecasting field. This combination of technologies means that anyone with access to a Python environment can quickly install a data catalogue, which will present them with Python objects, representing these large, distributed datasets. The user also gains access to standard post-processing methods that can serve as a benchmark reference to test against their developed algorithmic post-processing solutions. A description of each available dataset is provided below.

This paper is a first method of advertising this repository. A second step is to archive them on Pangeo (which is in the works). A third step is to engage NCAR, the UKMO, the EUMETNET working group on post-processing and NOAA in publicizing them as part of their recent initiatives in AI. For instance, they have been used in two recent EUMETNET workshops on post-processing and AI, and there is planned use in an NCAR 2021 Summer School. We will also promote use for student projects in regular university courses. We expect to track downloads, archive papers that come from the datasets and encourage researchers to communicate with the dataset owner and perhaps even write papers comparing AI techniques applied to these datasets. In keeping with our recommendations above, we will encourage documenting failures as well as successes to accelerate community learning.

### (a) Climate variability modes

We offer datasets representing two climate variability modes identified from eight separate operational weather forecast models for more than a decade worth of forecasts. These datasets are provided as benchmark datasets for training post-processing algorithms to improve forecasts of these large-scale modes of variability, and concomitantly, subseasonal forecast skill of other related weather patterns.

#### (i) MJO ensemble forecasts

**Figure 2.**
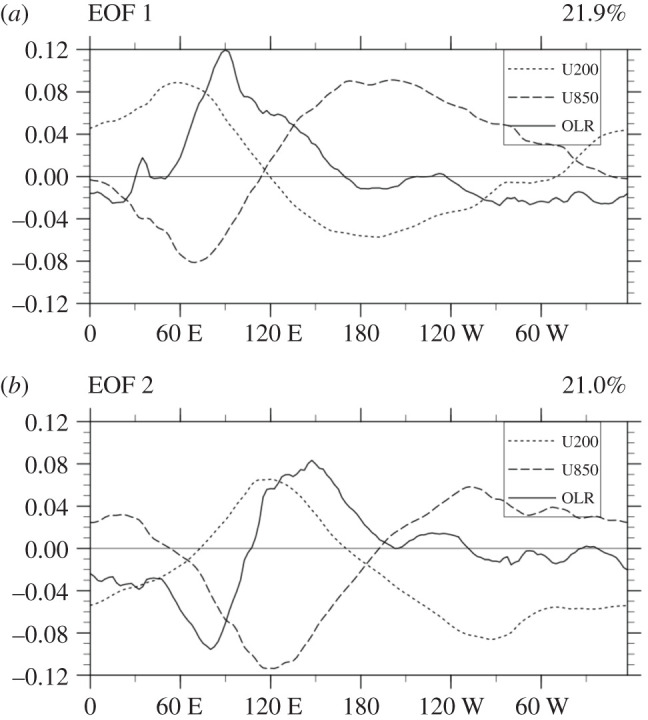
All-season multivariate (*a*) first and (*b*) second combined empirical orthogonal function (CEOF) modes of 20–100 day 15° S-15° N-averaged zonal wind at 850 hPa and 200 hPa from NCEP Reanalysis and OLR from the NOAA satellite for 1980–1999. The total variance accounted for by each mode is shown in parenthesis at the top of each panel. See Subramanian *et al.* [91,92] for a further exploration of the MJO.

The Madden-Julian Oscillation (MJO—[89,90]), a dominant intraseasonal mode of variability in the Tropics and a significant source of predictability globally on subseasonal timescales, has been identified using statistical techniques on forecast variables. We use the zonal winds at 850 hPa, 200 hPa, and outgoing longwave radiation from both the forecast models and observations to diagnose the MJO and evaluate its forecast skill. The dataset spans multiple ensembles (ranging from 51 to 10 members, depending on the operational weather forecast model) of daily forecasts from 2006 to 2019. Figure 2 represents the indices of the empirical orthogonal functions (EOFs) as a function of latitude for the coupled leading modes of the MJO.

#### (ii) PNA ensemble forecasts

Similarly, the Pacific North American pattern, which represents large-scale weather variability over the Pacific Northwest region, has been identified using the geopotential height field in a method consistent with Wallace & Gutzler [88] in both observations and model forecasts. The PNA is an important teleconnection pattern and heavily influences North American Weather. Additionally, the PNA is strongly forced by the El Nino-Southern Oscillation and its forecast skill is modulated likewise [93,94].

### (b) Global forecast system integrated vapour transport

**Figure 3.**
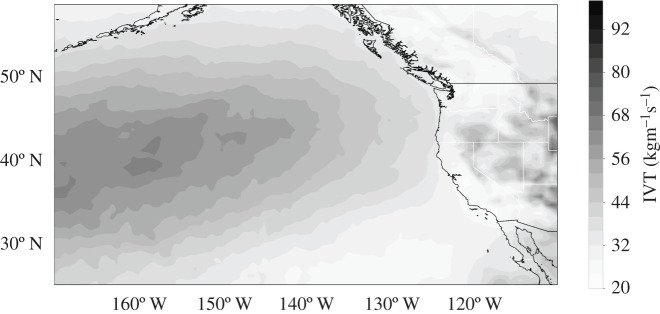
Root-mean-squared error of Global Forecast System's integrated vapour transport field 6 h forecasts issued 2006–2017. See Chapman *et al.* [68] for more detail.

A third dataset is the forecasted magnitude of integrated vapour transport (IVT) from the National Center for Environmental Predictions Global Forecast System (GFS). IVT is a combined momentum and thermodynamic metric that integrates specific humidity and *u* and *v* components of the wind speed from 1000 to 300 hpa. Predictions from the GFS [95] at a 0.5-degree horizontal spatial resolution on 64 vertical levels for daily 0000 and 1200 UTC model initializations are provided for this calculation. We present three forecast lead times of 6 h, 2 days and 1 week from 2006 to 2018. This includes approximately 8000 data fields for every forecast lead time or approximately 24 000 forecasted fields across all lead times. The region of interest spans coastal North America and the Eastern Pacific from 180° W to 110° W longitude, and 10° N to 60° N latitude. As a verifying observation field, we provide IVT from the National Aeronautics and Space Administration's Modern-Era Retrospective Analysis for Research and Applications version 2 (MERRA-2) reanalysis. MERRA-2 data are resolved on a 0.625 × 0.5 degree grid and interpolated to 21 pressure levels between 1000 and 300 hpa for IVT calculation [96,97]. For consistency, GFS predictions are then remapped to this grid resolution using a first- and second-order conservative remapping scheme. Further details can be found in figure 3 [68].

### (c) ECMWF Two-meter temperature ensemble over Germany

**Figure 4.**
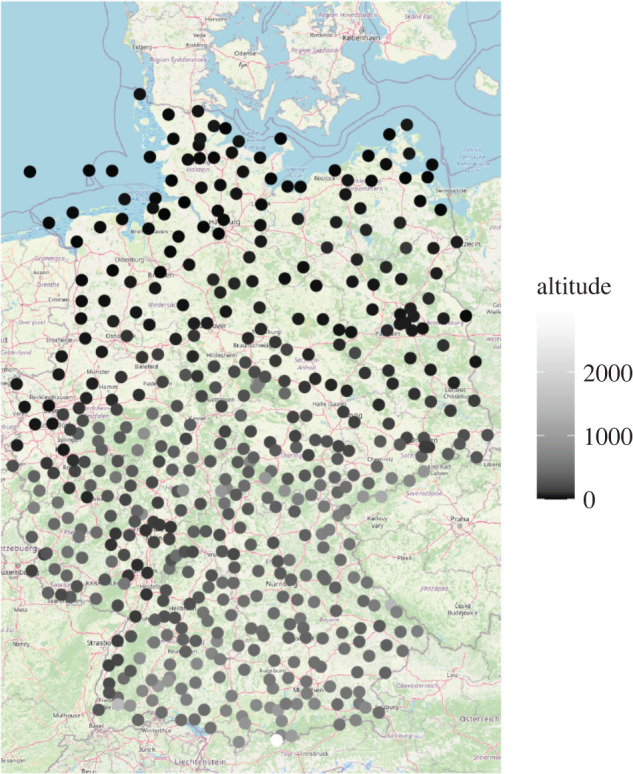
Station locations for the temperature dataset over Germany for the 2007–2015 training period. Shading of the dots indicates altitude. See [6] for more details. (Online version in colour.)

An example of short-range forecasts and verifying observations is a dataset of temperature observations at 537 stations over Germany and predictors derived from the ECMWF ensemble prediction system from 2007 to 2016. Predictors are the mean and standard deviation of 48-h ahead 50-member ECMWF ensemble forecasts of temperature and other variables, interpolated to station locations. The corresponding observations (valid at 00UTC) are obtained from surface synoptic observations stations operated by the German weather service. Details (including a list of predictors) are available in Rasp and Lerch [6]. Figure 4 indicates the locations and altitudes of the stations used for training.

### (d) UK surface road conditions

**Figure 5.**
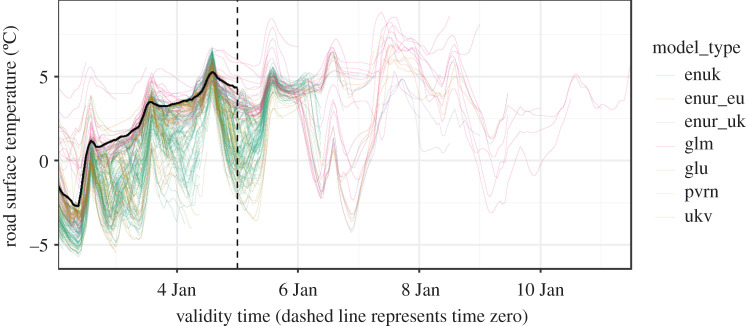
An example from the road surface temperature dataset. The solid black line shows observations up to ‘time zero’ (the vertical dashed line), beyond which various NWP forecasts (coloured lines) provide estimates of future outcomes. (Online version in colour.)

The fifth dataset contains numerical weather prediction forecasts from all models in the UK Met Office's Road Surface Temperature (MORST) forecasting system, along with corresponding road network temperature observations from Highways England. Data are provided for four random sites (location undisclosed) and spans 98 days from mid-December 2018 to late March 2019 on an hourly forecast lead basis from 0 to 168 h. Ground truth data are provided by the road surface temperature observed at the road network weather station for the concurrent forecast time. The dataset spans 2342 forecasting hours for each of the four sites. Spanning all lead times and owing to the fact that a multitude of forecasts are made for each hour by the time it is observed, the dataset spans over 1.34 million forecasts. This site-specific dataset highlights the challenges involved in providing fully probabilistic forecasts from NWP outputs. Kirkwood *et al.* [98] provide more details of the dataset and propose a machine learning-based solution to this forecasting problem. Figure 5 presents an example time series from this dataset.

### (e) Data archives

Our initiative to archive data in a repository to better enable testing AI methods is not unique. For instance, the Pangeo ecosystem (https://pangeo.io/) promotes open, reproducible and scalable science. The community provides documentation, develops and maintains the software and provides computing system architectures, focusing on open-source tools. It is in use by several national centres in meteorology, including the US National Center for Atmospheric Research (NCAR) and the UK Met Office (UKMO), as well as The Alan Turing Institute (the UK's national institute for data science and artificial intelligence) and the British Antarctic Survey (BAS), among others. Note that parallel data archive efforts are underway in other communities, including Environnet [99], Weatherbench [100] (https://arxiv.org/abs/2002.00469), Spacenet (https://arxiv.org/abs/1807.01232) and various authors who make their datasets public [71] among others.

## 6. Concluding thoughts

Post-processing weather and climate output using AI engenders an active and well-established community that has already provided a host of research demonstrating value for weather forecasting. In that sense, it is the most mature sector of machine learning and artificial intelligence used in the weather and climate community. This conference review was framed around the conversations between machine learning and post-processing experts; we have focused on the future impact of pursuing modern machine learning techniques and what it would look like to successfully implement these methods widely.

We have set in motion a call to action to further explore modern machine learning techniques and their applicability in the weather and climate communities. We hope to inspire further study and resources to be dedicated to model improvement through post-processing.

Specifically, we call for 1) development of a data repository for fast development of post-processing techniques, 2) data standardization methods (FAIR), 3) studies on interpretability methods, 4) metadata and model documentation for labelled training data and 5) a database of recorded AI failures to limit any duplication of effort across the research community.

An actionable outcome of this effort is the initialization of a repository beginning with five datasets that represent an interesting range of weather and climate problems, both deterministic and probabilistic, to test AI methods [86]. In addition, we have provided Jupyter notebooks to aid processing these datasets and comparing them to a documented baseline. The authors invite the readers to test their own methods on these datasets and contribute additional interesting datasets to this archive.

The issues brought forth here suggest a roadmap for AI to become ubiquitous in post-processing weather and climate model output. Specifically, a first step is initiating repositories such as the one offered here, together with a set of notebooks and datasets to standardize testing new methods. These repositories can be advertised and promoted, such as in this paper and through workshops and courses, such as those offered by the institutions represented by the coauthors of this paper. Offering a dedicated website and portal to facilitate benchmarking, collaboration and publication of the results, including negative results to assure that time is best leveraged. Through making such datasets available, promoting the FAIR principals, and encouraging full use of these methods, we expect that AI will continue to expand and become a yet more necessary component of weather and climate prediction.

## Data accessibility

The datasets described in §5 are stored at https://doi.org/10.6075/J08S4NDM. Jupyter notebooks to process those data and to evaluate methods using the data are available at https://github.com/NCAR/PostProcessForecasts.
